# Improved Discrimination of Tumors with Low and Heterogeneous EGFR Expression in Fluorescence-Guided Surgery Through Paired-Agent Protocols

**DOI:** 10.1007/s11307-021-01656-3

**Published:** 2021-10-14

**Authors:** Cheng Wang, Xiaochun Xu, Margaret Folaron, Jason R. Gunn, Sassan Hodge, Eunice Y. Chen, P. Jack Hoopes, Kenneth M. Tichauer, Kimberley S. Samkoe

**Affiliations:** 1 Thayer School of Engineering, Dartmouth College, Hanover, NH, USA; 2 Department of Surgery, Dartmouth-Hitchcock Medical Center, Lebanon, NH, USA; 3 Geisel School of Medicine, Dartmouth College, Hanover, NH, USA; 4 Biomedical Engineering, Illinois Institute of Technology, Chicago, IL, USA 2021

**Keywords:** Paired-agent imaging, Fluorescence-guided surgery, Head and neck squamous cell carcinoma, Epidermal growth factor receptor, ABY-029, IRDye 700DX

## Abstract

**Purpose:**

The goal of fluorescence-guided surgery (FGS) in oncology is to improve the surgical therapeutic index by enhancing contrast between cancerous and healthy tissues. However, optimal discrimination between these tissues is complicated by the nonspecific uptake and retention of molecular targeted agents and the variance of fluorescence signal. Paired-agent imaging (PAI) employs co-administration of an untargeted imaging agent with a molecular targeted agent, providing a normalization factor to minimize nonspecific and varied signals. The resulting measured binding potential is quantitative and equivalent to *in vivo* immunohistochemistry of the target protein. This study demonstrates that PAI improves the accuracy of tumor-to-healthy tissue discrimination compared to single-agent imaging for *in vivo* FGS.

**Procedures:**

PAI using a fluorescent anti-epidermal growth factor receptor (EGFR) affibody molecule (ABY-029, eIND 122,681) with untargeted IRDye 700DX carboxylate was compared to ABY-029 alone in an oral squamous cell carcinoma xenograft mouse model at 3 h after dye administration (*n* = 30).

**Results:**

PAI significantly enhanced tumor discrimination, as compared to ABY-029 alone in low EGFR-expressing tumors and highly heterogeneous populations including multiple cell lines with varying expression (diagnostic accuracy: 0.908 vs. 0.854 and 0.908 vs. 0.822; and ROC curve AUC: 0.963 vs. 0.909 and 0.957 vs. 0.909, respectively) indicating a potential for universal FGS image thresholds to determine surgical margins. In addition, PAI achieved significantly higher diagnostic ability than ABY-029 alone 0.25–5-h post injection and exhibited a stronger correlation to EGFR expression heterogeneity.

**Conclusion:**

The quantitative receptor delineation of PAI promises to improve the surgical therapeutic index of cancer resection in a clinically relevant timeline.

## Background

Completeness of surgical resection is a critical determinant for the survival of patients with head and neck cancers. Positive tumor margins in oral cavity tumors increases tumor-related death at 5-years by 90 % compared to those with truly negative margins [1], but the use of wide margins to remove residual tumor in the head and neck region can lead to severe morbidity. The near ubiquitous overexpression of epidermal growth factor receptor (EGFR)—with estimates of > 90 % overexpression in squamous cell carcinoma (SCC) [2, 3]—has led to the development of numerous molecular therapeutic agents, which have been subsequently leveraged for fluorescence imaging [4–8]. The goal of molecular-targeted fluorescence-guided surgery (FGS) is to improve the surgical therapeutic index based on the overexpression of the molecular target in tumor compared to normal tissue. Several studies have reported advantages of FGS for identifying bulk tumor and tumor margins using therapeutic antibodies (cetuximab, panitumumab) labeled with IRDye 800CW (LI-COR Biosciences, Inc.) [7, 9–11]. However, true molecular contrast using FGS is confounded by heterogeneous uptake and nonspecific retention of targeted imaging agents within all tissue types. Paired-agent imaging (PAI) methods have the potential to overcome these confounding effects through co-administration of a second, untargeted, control fluorescent agent enabling imaging of the receptor concentration, rather than agent concentration [12]. This preclinical project compares the accuracy of tumor discrimination using conventional “single-agent imaging” (SAI) and a proposed PAI strategy in an orthotopic xenograft mouse model of human head and neck cancer.

In recent years, we have advanced two initiatives to improve FGS: the aforementioned PAI and the development of an anti-EGFR fluorescent affibody molecule (ABY-029). PAI, which reports the “binding potential” (BP, a value proportional to receptor concentration), has been used in a variety of EGFR-overexpressing xenograft cell lines to demonstrate that tumor-averaged binding potential scales linearly with EGFR both *in vivo* and ex vivo [13]. This *in vivo* phenomenon was linearly correlated with ex vivo tumor EGFR immunohistochemistry [14] and shown to noninvasively detect fewer than 200 tumor cells in draining lymph nodes [15]. However, the ability of PAI to truly improve tumor discrimination in FGS has never been quantitatively assessed.

ABY-029, an affibody dye conjugate, has been developed to reduce administration-to-imaging time (hours instead of days) and reduce immunogenicity compared to antibody imaging agents [6]. ABY-029 is currently being tested in phase 0 studies at Dartmouth College in a number of solid tumor types, including head and neck cancers (NCT 03282461). In the work presented here, we utilize two orthotopic SCC base-of-tongue tumors (FaDu and Detroit 562) and a highly expressing EGFR SCC of the skin (A431) to compare the accuracy and efficiency of FGS tumor resection using ABY-029 alone versus PAI (the latter a combination of ABY-029 with “control” IRDye 700DX carboxylate).

## Methods

### Cell Lines and Culture Methods

Human squamous cell carcinoma cell lines used in this study included FaDu, a pharynx carcinoma; Detroit 562, a metastatic pharynx carcinoma derived from pleural effusion; and A431, an epidermal SCC. All three cell lines were purchased from the ATCC (Manassas, VA, USA) and were cultured according to ATCC specifications with the addition of 1 % penicillin–streptomycin.

### Imaging Agents

ABY-029 was obtained from the University of Alabama at Birmingham (UAB) Vector Production Facility and manufactured under Good Laboratory Practice (GLP) as previously described [6]. The ABY-029 human microdose is defined as 30 nmol per human, (3.96 μg/kg for a 60-kg human). Using the method of Reagan-Shaw [16], the mouse-equivalent microdose was determined to be 48.8 μg/kg for an average 22-g mouse, for a final dose of 1.07 μg/mouse. IRDye 700DX NHS ester was purchased from LI-COR Biosciences, Inc. (Lincoln, NE) and converted to carboxylate form by dissolving in PBS (pH = 8.5) and stirring at room temperature for 5 h.

### Mouse Xenograft Model

All animal procedures were approved by the Dartmouth Institutional Animal Care and Use Committee (IACUC) and conducted according to NIH-OLAW and AAALAC guidelines. Female, athymic nude mice, 6–8 weeks of age, were purchased from Charles River Laboratories (Wilmington, MA). Tongue tumors were implanted using a 25-gauge needle to implant 5 × 10^5^ cancer cells in 50 μl of culture medium. Each cell line was implanted in ten mice (*n*_total_ = 30). PAI was performed on six of the ten mice with FaDu tumors, and seven of the ten mice with Det 562 tumors and with A431 tumors; the remaining ten mice served as controls to quantify effects of autofluorescence. The additional 30 mice were implanted with FaDu for an administration-to-imaging time study (*n* = 5 mice/time point). Tumor implantation success rate was 100 %, and all imaging was carried out when tumors had a diameter between 3 and 4 mm.

### ABY-029 and IRDye 700DX Fluorescence Imaging

**Fig. 1.**
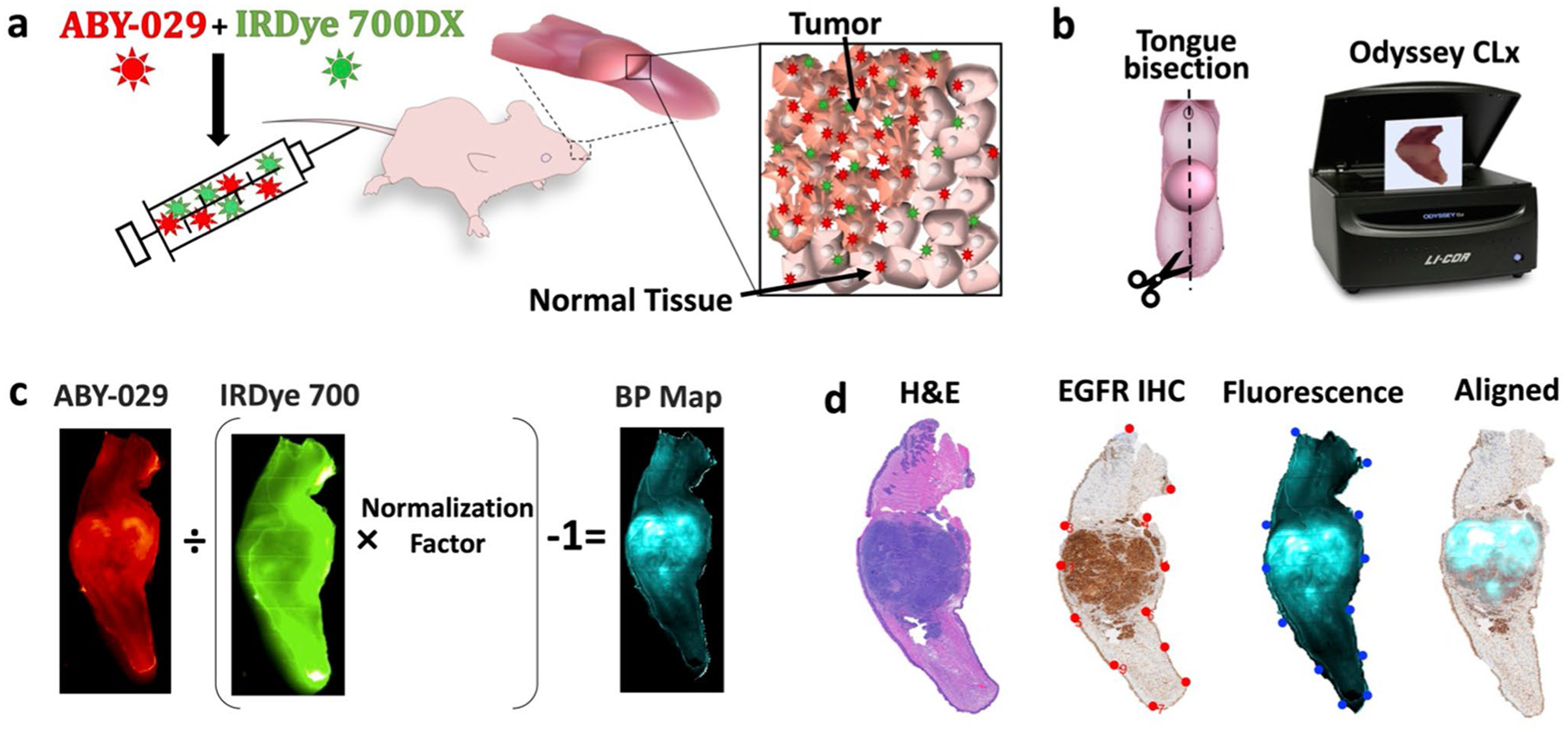
Schematic of PAI experimental and computational procedures. **a** Mice, with xenograft murine tongue tumors, were administrated ABY-029 and IRDye 700DX by tail vain injection. *Inset*—a pictorial representation of the paired-agent distribution 3 h after administration, where both agents are present due to nonspecific binding and uptake in all tissues, while only ABY-029 specifically bound to receptors. **b** After sacrifice, the tongue is removed and bisected. Tumor and normal tissue fluorescence were imaged using Odyssey CLx in the 700-nm and 800-nm channels. **c** Binding potential (BP) maps representing available EGFR concentrations were calculated by using single time point model. **d** For further impartial analysis, pathological and fluorescence images were processed by landmark co-registration.

Mice were administered 200 μl of a 1:10 molar ratio of 0.68-μM ABY-029 and 6.8-μM IRDye 700DX in sterile phosphate-buffered saline (PBS) via intravascular tail vein injection (Fig. 1a). Injection concentrations were selected to ensure > 6:1 fluorescence signal-to-background at 3-h post-injection (Supplemental Data, Section S2). The mice were euthanized by cervical dislocation while anesthetized to a surgical plane (1.5–2 % isoflurane, 1 l/min O_2_) at 3-h post-imaging agent administration, with the exception of the time study where euthanasia was carried out at 0.25, 0.5, 1, 2, or 5 h, depending on the mouse group. Note that the 3-h FaDu tumors were also used in the time study to minimize animal use. Following euthanasia, tongues were excised at the base, bisected along the midline raphe, and placed on a glass slide cut-face down. Ex vivo images of the tissues were collected for both ABY-029 and IRDye 700DX carboxylate on the Odyssey CLx (LI-COR Biosciences, Inc.) using the following settings: auto function for laser intensity, 1-mm focus offset, medium quality, and 42-μm resolution.

### PAI Binding Potential Map Creation

PAI binding potential (*BP*) maps were calculated from of the ABY-029 and IRDye 700DX images using the single time point (STP) method (Eq. 1), first described by Tichauer et al. [17] (Fig. 1b). Note that in this previous work, pre-injection images were subtracted from the post-injection image to remove the contribution of autofluorescence.

Pre-administration images were not available in this study, and autofluorescence contribution is discussed in the Supplemental Data, Section S2. For each pixel within the image, the BP was calculated using

(1) $$BP=\frac{I_{T}}{I_{U}NF}-1$$

where *I*_T_ and *I*_U_ are the pixel intensity of the targeted (ABY-029) and untargeted (IRDye 700DX) imaging agents, respectively, and *NF* is the normalization factor determined by Eq. 2.

(2) $$NF=\left(BP_{tip}+1\right)\frac{{\bar{I}}_{T(\text{norm})}}{{\bar{I}}_{U(\text{norm})}}=\frac{1.5{\bar{I}}_{T(\text{norm})}}{{\bar{I}}_{U(\text{norm})}}$$

where ${\bar{I}}_{T(\text{norm})}$ and ${\bar{I}}_{U(\text{norm})}$ are the mean pixel intensities of the targeted and untargeted imaging agents, respectively, in the normal tongue and *BP*_tip_ is the binding potential in the tip of the tongue, which was artificially set to 0.5 in order to have a “near-zero” BP value in the normal tongue tissue while avoiding negative pixels. The *NF* was calculated independently in every mouse. The selection of this signal normalization protocol is described in detail in the Supplemental Data, Section S3, where it is demonstrated that the methodology does not alter the detection metrics.

### Pathology

After imaging, the tongue sections were placed on filter paper to maintain orientation and fixed in 10 % buffered formalin (Biochemical Sciences, Inc.) in histological cassettes. Standard hematoxylin and eosin (H&E) and EGFR immunohistochemistry (IHC) staining were performed by the Norris Cotton Cancer Center Pathology Translational Research Shared Resource as described previously [14]. RGB images of whole H&E and EGFR IHC tissue sections were collected on the Vectra 3 (PerkinElmer) at 4 × magnification. The image was saved as an RGB three-image stack .qptiff file and then converted to a single .tiff file using the concatenate arrays function (*cat*) in MATLAB version R_2017a.

### Image Preparation and Co-registration

Five image types were used in this study: H&E, EGFR IHC, IRDye 700DX (untargeted agent), ABY-029 (targeted agent), and BP maps. Prior to co-registration, several steps were taken to prepare the images. ABY-029, IRDye 700DX, and BP maps were inherently co-registered as a function of imaging on the Odyssey CLx (42 μm/pixel). The H&E and EGFR IHC images collected on the Vectra scanner (1 μm/pixel) were resized to match the fluorescent images. The brown stain indicating EGFR in the IHC images was isolated by using the H DAB Color Deconvolution script in FIJI [18] and then normalized to the average stain intensity in the placenta positive control slide for each staining batch to correct for variations in stain intensity due to color development. Image co-registration of the fresh tissue sections with pathology was performed using a previously described procedure [19]. Briefly, the BP map was co-registered to the H&E and EGFR IHC image using *warp_it* in MATLAB, which utilizes point matching to spatially transform and register the images. Visualization of the overlaid images is provided in the Supplemental Data, Section S4.

### Image Analysis and Statistics

For each sample, a histopathologist (author SH) drew regions of interest (ROI) for normal tongue muscle, tumor, and salivary gland in H&E images, which were co-registered by the methods described in fluorescence and BP images for both mean and pixel-by-pixel analysis of tissue types. The visualization of ABY-029, IRDye 700, and BP are presented by “fire,” “kryptonite,” and “teals” pseudo colormaps as defined by *COLORMAP* (https://jdherman.github.io/colormap/). Histograms and receiver operating characteristic (ROC) curves were created in MATLAB. Diagnostic parameters, including area under the curve (AUC), sensitivity, specificity, accuracy, positive predictive value (PPV), and negative predictive value (NPV), were determined. Statistically significant differences in group means were analyzed in Prism 8 (GraphPad) using a one-way ANOVA with Bonferroni correction to avoid type I error. Pearson’s correlation coefficient was used to measure the correlation of both EGFR IHC and fluorescent images to BP maps. The least squares line was fit in scatter plots of intensity. To study the impact of image resolution on the correlation of EGFR staining and fluorescent images, *image-pyramid* in MATLAB was used to decrease image resolution by averaging four adjacent pixels. Contrast-to-variation ratio (CVR) was defined by:

(3) $$CVR=\frac{\mu \left(I_{T}\right)-\mu \left(I_{N}\right)}{\sqrt{\sigma_{T}^{2}+\sigma_{N}^{2}}}$$

*μ*(*I*_*T*_) and *μ*(*I*_*N*_) represent mean fluorescence or BP, and σT and σN represent the standard deviation of fluorescence or BP values in tumor and normal tissue, respectively.

## Results

### Administration Dose and the Normalization Factor

**Fig. 2.**
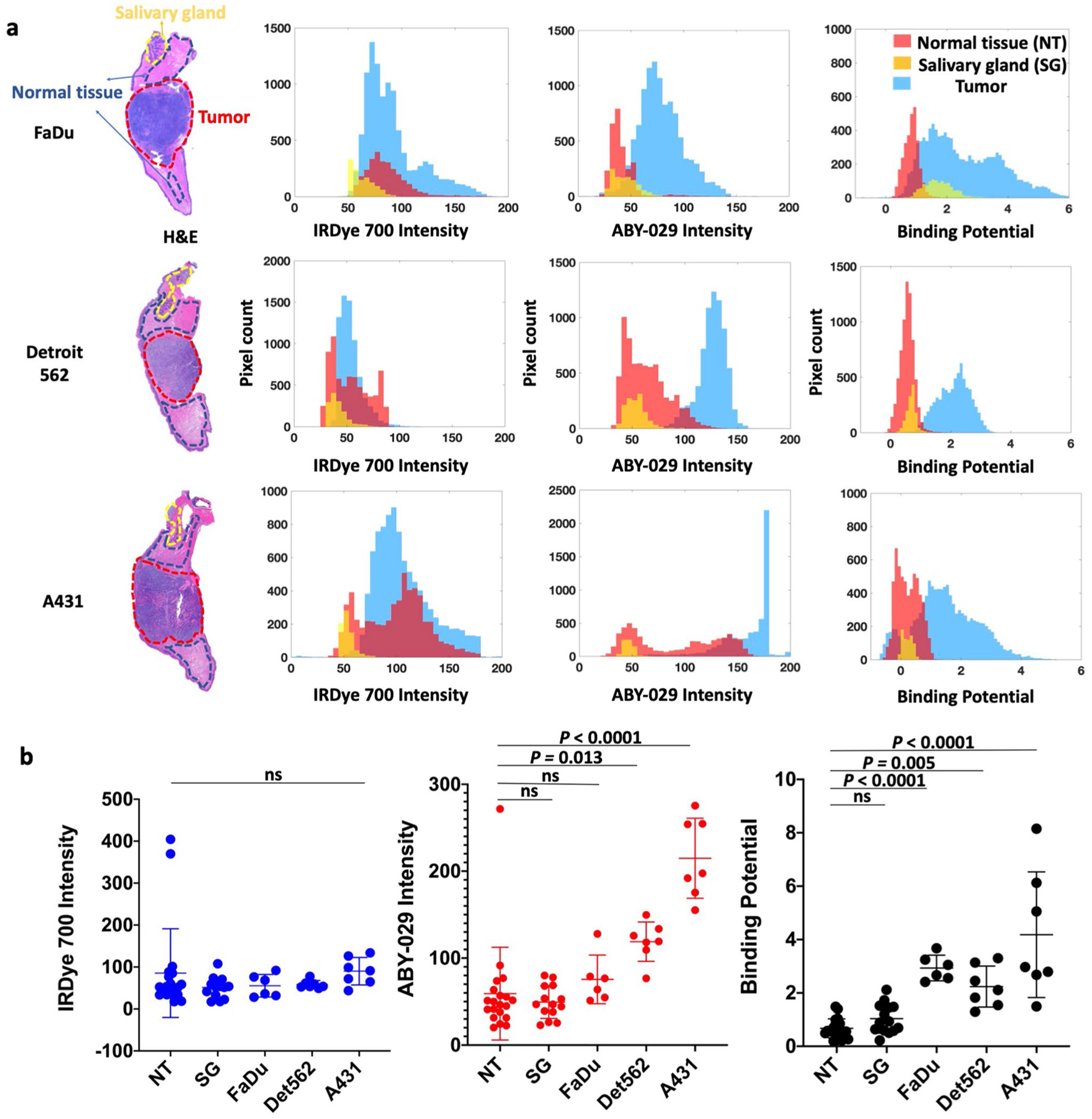
Tumor and normal tissue discrimination by signal intensity. **a** After image co-registration, pathologist-defined ROIs of normal tongue (NT), salivary glands (SG), and tumor were drawn on the gold standard H&E images. Signal intensity in the ROIs on corresponding untargeted fluorescence, targeted fluorescence, and BP images are compared for a representative tumor from each cell line. **b** The average signal from each ROI was plotted for all animals in three tumor groups. For clarity, only the statistical mean comparison with normal tissue is shown.

In previous studies [14, 15], a pre-injection background image was collected and used to remove the absolute tissue autofluorescence signal on a pixel-by-pixel basis; however, in this study and during a typical FGS process, pre-injection images within the excised tissue and/or surgical wound bed are not available. This had two effects on the study design. First, an understanding of typical autofluorescence levels in our samples was required to ensure that appropriate concentrations of fluorescent imaging agents were administered to yield “negligible” (> 6:1 fluorescence:autofluorescence) levels of autofluorescence. A 1:10 molar ratio of ABY-029:IRDye 700DX was used in order to obtain fluorescence signal ~ 6–8 times the autofluorescence at 3 h (see Fig. 2b and Supplemental Figure S2). Second, the normalization factor (NF) was determined at each time point by setting the BP equal to 0.5 using the average ABY-029 and IRDye 700DX fluorescence signal in the tip of the tongue, rather than using EGFR-devoid leg muscle in the first post-administration image. CVR (Eq. 3) was used to standardize the measurements and compare image contrast between PAI and SAI as tumor-to-background ratios (TBR) were found to be unstable (see Supplemental Figure S5).

### Discrimination of Tumor and Normal Tissue

To evaluate the ability of PAI and SAI to distinguish tissue types based on signal alone, we analyzed the resultant images into two ways: region of interest (ROI) averages and region pixel-to-pixel comparisons. After co-registration, the fluorescence intensity of ABY-029 and the BP were compared for tumor (FaDu, Detroit 562, and A431), normal tongue, and salivary gland. A representative example of fluorescence intensity and BP is plotted in Fig. 2a to demonstrate the variability of the signal in each tissue. The ROI-specific averages for each tissue type are shown in increasing order of EGFR expression (Fig. 2b). The tumor cell line EGFR expression was determined using quantitative flow cytometry (see Supplemental Data, Section S5). A one-way ANOVA analysis with Bonferroni correction demonstrated significant differences between tumor and normal tissues for mean ABY-029 fluorescence (*p* < 0.0001) and BP (*p* < 0.0001), but no significant differences for IRDye 700DX (*p* = 0.543). Individual Bonferroni mean comparison indicates that all three tumor lines are different from normal tongue when considering PAI determined BP (*p* ≤ 0.005), but only the high and moderate expressing tumors lines (A431 and Detroit 562, respectively) are different for ABY-029 fluorescence intensity (*p* ≤ 0.01). When compared to normal salivary glands, the average PAI determined BP is not significantly different for any of the tumor lines, while the average ABY-029 fluorescence intensities for A431 and Detroit 562 are (*p* ≤ 0.01), but FaDu is not.

**Fig. 3.**
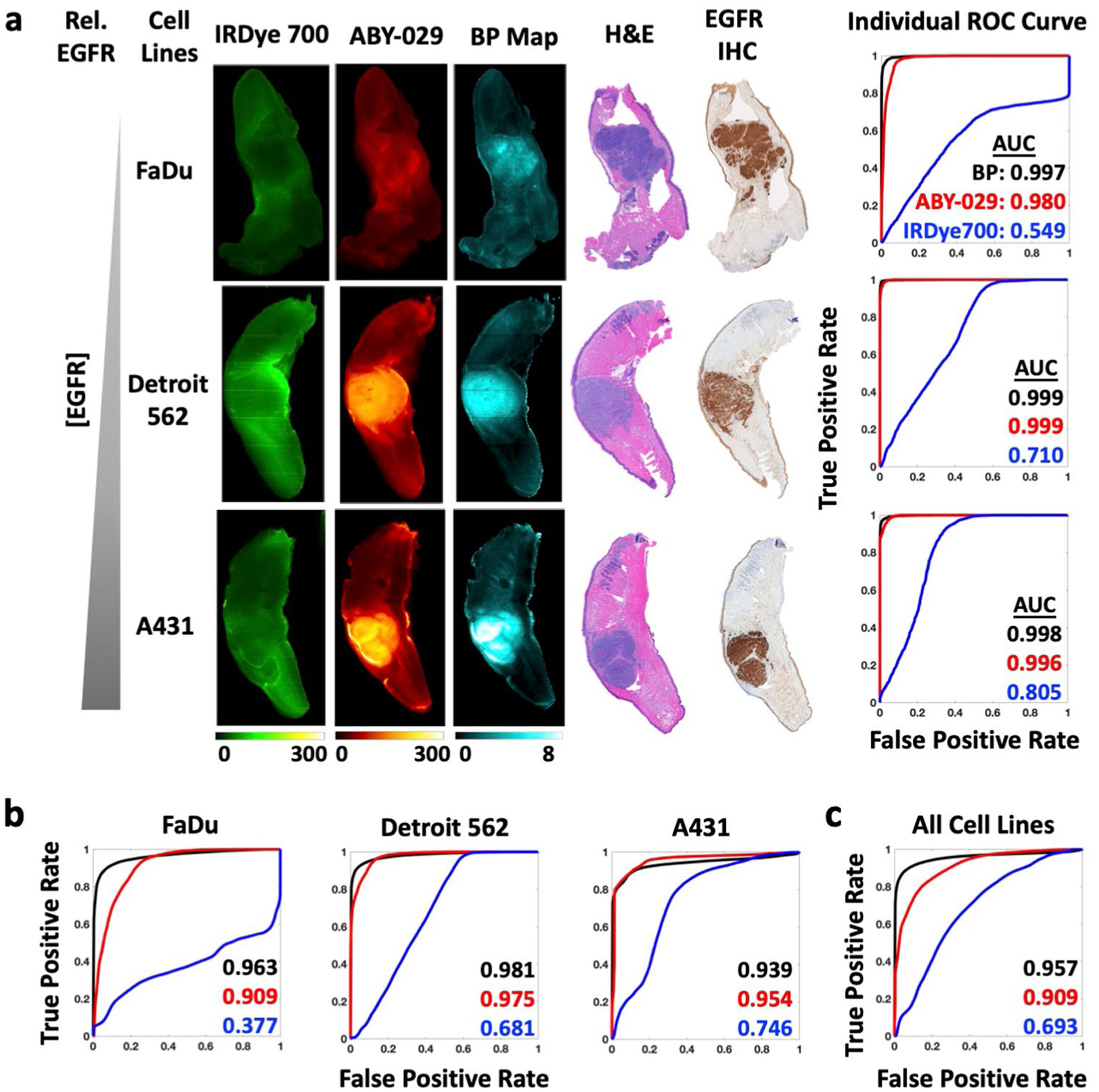
Pixel-by-pixel analysis demonstrates PAI has higher diagnostic accuracy than SAI. **a** ROC curve analysis was performed for IRDye 700DX, ABY-029, and BP using H&E as the gold standard. **b** Cohort ROC data for individual tumor type, FaDu, Detroit 562, and A431 (*n* = 6 or 7) and **c** all tumor groups mixed together for varied EGFR-expressing population (*n* = 20), demonstrate that BP maps have higher AUC compared to ABY-029 alone.

Pixel-wise histograms presented in Fig. 2a demonstrate a varying amount of overlap in the distribution of pixel values between normal and tumor groups indicating the potential for misclassification. Therefore, comparison of SAI and PAI for each tissue type was performed on the co-registered images on a pixel-to-pixel basis (Fig. 3). Representative co-registered IRDye 700DX fluorescence, ABY-029 fluorescence, and BP map images are shown for each tumor line with pathological images (Fig. 3a). Pixel intensities from fluorescence images and BP maps were used to plot receiver operating characteristic (ROC) curves to evaluate the diagnostic ability of SAI and PAI. Although BP maps yield slightly higher area under the curve (AUC) values than ABY-029 alone in the representative samples, this result was not statistically significant on the single animal level. The BP AUCs evolved from average individual ROC curve of 0.971, 0.982, and 0.953 to cohort ROC curve of 0.963, 0.981, and 0.939, for FaDu, Det 562, and A431, respectively, and then to all cell lines ROC curve of 0.957, while ABY-029 AUCs varied at a larger scale from 0.926, 0.991, and 0.910; to 0.909, 0.975, and 0.954; and then to 0.909 (Fig. 3). The statistical analysis shows that ROC curves between PAI and SAI have *p* value less than 0.001 in individuals, cohorts, and all cell lines. The cohort diagnostic accuracy statistics are summarized in Table 1, with the higher value highlighted in green for ease of interpretation. BP maps demonstrated higher specificity, positive predictive value (PPV), and higher diagnostic accuracy, in all tumor lines with the exception of A431 (the highest EGFR-expressing cell line of the group studied).

### Representation of Tissue EGFR Expression Heterogeneity

**Fig. 4.**
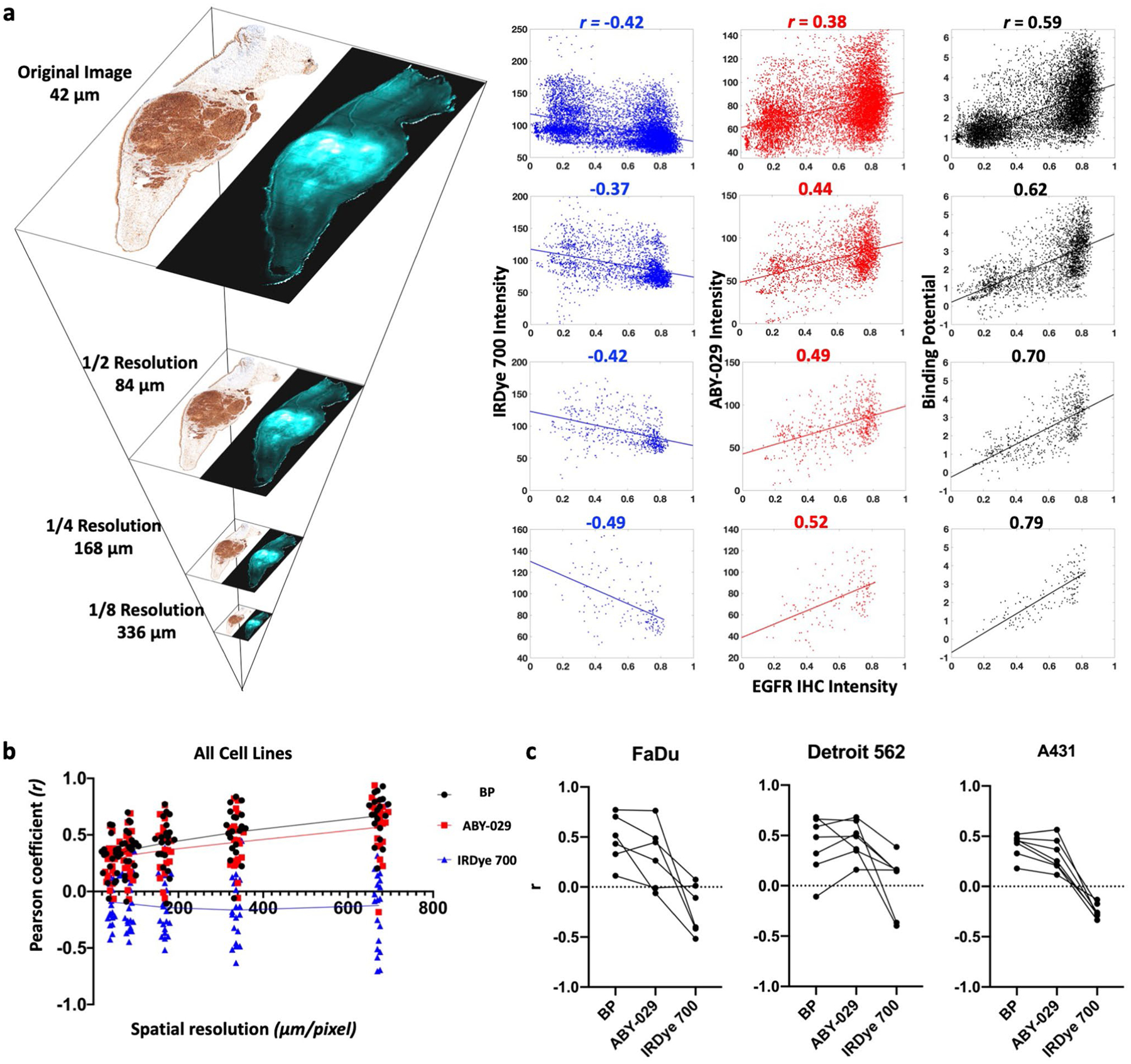
BP and fluorescence images were correlated to EGFR IHC on a pixel-to-pixel basis using the Pearson correlation coefficient (*r*). EGFR expression heterogeneity is most accurately represented by PAI determined BP. **a** Pixel resolution was reduced to the clinically relevant range by the image pyramid method. BP and fluorescence against IHC intensity were plotted for representative images at four image resolutions. **b** The *r* from all specimen were plotted against spatial resolution, which indicates a steady increased correlation to IHC for BP and ABY-029. **c** Pearson’s correlation coefficients of BP, ABY-029, and IRDye 700 with IHC at 168-μm image resolution were presented in three panels, with each line representing data from individual mouse.

EGFR expression within tumors was highly heterogeneous, especially compared to normal tissues, as can be observed in the IHC images (Fig. 3 and Supplemental Figure S2 & S3). Heterogeneous EGFR expression can contribute to difficulties in distinguishing tissues; therefore, we assessed the pixel-by-pixel linear correlation between IHC stain intensity with BP, ABY-029, and IRDye 700DX fluorescence (Fig. 4). To assess the effects of co-registration error on the high-resolution (42 μm/pixel) images, an image pyramid algorithm (Fig. 4a) was used to incrementally decrease resolution. The resulting scatter plots and the corresponding Pearson coefficients (*r*) for each resolution tested in a representative FaDu tumor are shown in Fig. 4a. At 42 μm/pixel, BP demonstrated a strong correlation (*r* between ± 0.50 and ± 1) with IHC, while IRDye 700DX and ABY-029 exhibited moderate (*r* between ± 0.30 and ± 0.49) negative and positive correlations, respectively. The change of *r* with decreasing image resolution in all specimen is plotted in Fig. 4b. As image resolution decreased, the strength of the correlation between EGFR IHC and all three image types increased, with BP maintaining the strongest correlation with EGFR IHC at each level. Clinical imaging systems (wide-field to endoscopic) have spatial resolutions of 50–500 μm [20]. Therefore, Pearson’s coefficients of individual mouse in 1/4 reduction of the original resolution (168 μm) were plotted in each tumor group. Overall, the average Pearson coefficients were 0.4 ± 0.2, 0.4 ± 0.2, and −0.1 ± 0.3 for BP, ABY-029, and IRDye 700DX, respectively.

### Reduction of Administration-to-Imaging Time

**Fig. 5.**
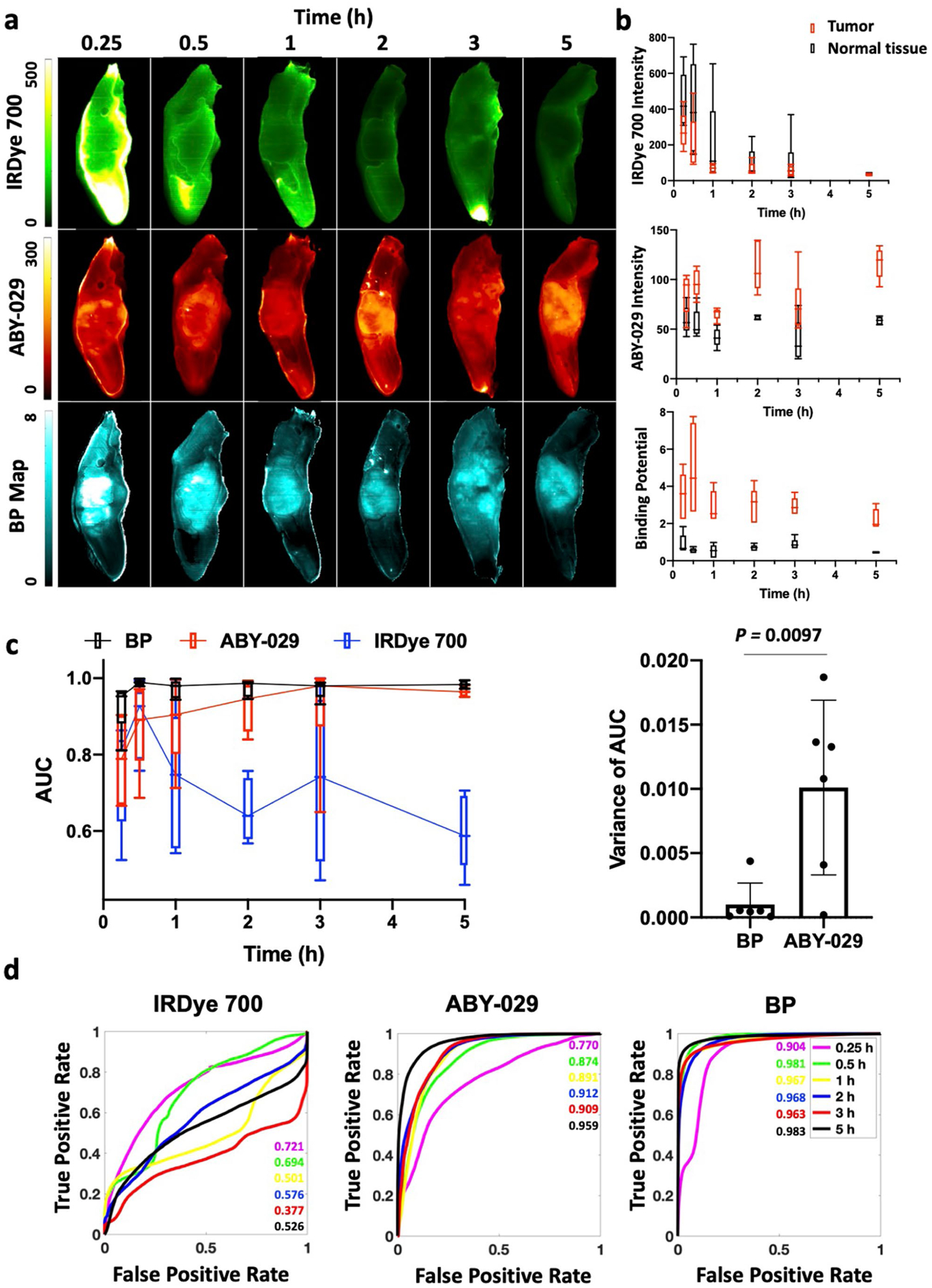
Comparison of administration-to-imaging time for PAI and SAI. **a** Representative images of the xenograft FaDu model are shown for SAI and PAI at each time point. **b** The average signals of tumor and normal tongue for each time point were graphed. BP is fairly constant over the 5-h period, while fluorescence signal decreases for IRDye 700DX and increases for ABY-029 over time. **c** ROC analysis indicates diagnostic abilities of ABY-029 and BP improve over time; however, BP outperforms ABY-029 images, especially at short administration-to-imaging time points.

To maximize observed FGS contrast, the delay time between agent administration and surgery must be optimized. To study administration-to-imaging time of PAI, mice (*n* = 5 or 6 per group) were co-administered ABY-029 and IRDye 700DX and then euthanized at varying time points up to 5 h after administration (Fig. 5). Representative SAI and PAI images for a single animal at each time point (Fig. 5a), as well as boxplots of the signal intensity in the tumor and normal tongue regions over all times, are shown (Fig. 5b).

Individual AUCs of ROC and the variances of AUCs at each time point were plotted in Fig. 5c. The variance of the PAI AUC and SAI AUC are significantly different with average and standard deviation of 0.001 ± 0.002 and 0.010 ± 0.007, respectively. Same analysis of CVR also is performed, but no significance is found (Supplemental Figure S4).

ROC curves for three imaging methods were plotted for each time point. The AUCs of both BP and ABY-029 alone increased over time while no trend of IRDye 700 AUCs with increased time was observed (Fig. 5d). At every time point, the PAI AUCs were significantly higher than SAI ABY-029 AUCs (*p* < 0.00001). Moreover, SAI ABY-029 AUC increased over the 5-h administration-to-imaging time with no demonstration of stabilization in signal, while PAI exhibited higher and more consistent AUCs over time.

## Discussion

Molecular PAI protocols have been proven to provide significant advantages for estimating true molecular contrast and for enabling unmatched specificity and sensitivity [14, 15]. Here, we demonstrate that average PAI BP intensities for tumors with varying EGFR expression were statistically higher than those in normal tongue (*p* ≤ 0.005). Comparatively, the average SAI fluorescence intensity was significantly larger in tumors with high (A431) or moderate (Detroit 562) EGFR-expressing tumors, while FaDu, the lowest EGFR-expressing cell line utilized, was found to have the same average fluorescence intensity as normal tissues (Fig. 2). However, the broad signal variance of both BP and fluorescence intensity in PAI and SAI, respectively, warranted further investigation of the diagnostic abilities of these methodologies (Fig. 2a). Therefore, studies mimicking in-patient and back-table (ex vivo) intraoperative assessment strategies [2] were undertaken to compare PAI and SAI to gold standard tumor delineation techniques using H&E and EGFR immune-stained formalin-fixed paraffin-embedded pathology, with co-registration to PAI and SAI and correlation on a pixel-to-pixel basis (Fig. 3). In an ideal imaging protocol, the autofluorescence signal would be subtracted from a pre-injection image. The difference in the PKs of the two imaging agents would be eliminated by using a deconvolution method that was developed and tested in preclinical study [12]. Due to the incapability of acquiring autofluorescence and dynamic curves of imaging agents in clinical setting, single time point BP model was used here with compromise.

In 1998, Grandis et al. demonstrated that the EGFR expression in human HNSCC was highly varied with a range of 5–233 % expression as compared to the “gold standard” A431 cell line [2]. In Fig. 3 and Table 1, we demonstrate using AUC of ROC curves and diagnostic tests, that PAI can distinguish tumor from normal tissue with higher accuracy than SAI in cases of low EGFR expression (FaDu, 6.2 % of A431 expression—see Supplemental Material Section S5) and in populations with high individual variance (all cell lines, nearly two-orders of magnitude difference in EGFR expression). For the relatively moderate EGFR-expressing tumor line, Detroit 562, PAI outperformed SAI in all categories except sensitivity and was only narrowly better in negative predictive value (NPV). Interestingly, in the high EGFR-expressing cell line (A431), the two techniques were comparable, with SAI outperforming PAI for AUC, sensitivity, accuracy, and NPV. Both sensitivity and NPV include “false negatives” in the denominator, suggesting that Detroit 562 and A431 had a high number of pixels within the pathologist-designated tumor ROIs that were classified as normal tissue based on PAI as compared to SAI. This discrepancy can be explained by the fact that the “pathologist-determined tumor” contains regions of non-EGFR–expressing tissue, and PAI is designed to enhance contrast as a function of targeted molecule (in this case, EGFR expression). When considering the whole tumor on a pixel-to-pixel basis, the regions devoid of EGFR decreased the measured predictive power of PAI because it is truly a molecular signal, unlike SAI, which is a summation of molecular targeted and nonspecific signal from the enhanced permeability and retention (EPR) effect [21]. Data in soft-tissue sarcomas suggests this is the case by demonstrating that overall tumor contrast was enhanced and fluorescent signal variance minimized by simultaneously imaging perfusion-based ICG accumulation with ABY-029 in a single imaging channel [22, 23]. However, perfusion agents may be better for this capacity than targeted agents like ABY-029. Further investigation is required in tumors with large negative regions or with lower cellular density—often seen in aggressive cell lines commonly used for xenograft models (due to fast growth rate), yet not necessarily indicative of patient population tumor characteristics.

The ability of PAI to distinguish low EGFR-expressing populations and highly heterogeneous EGFR expression populations from normal tissue is important for surgical guidance for several reasons. First, this suggests that PAI may be more sensitive to micrometastases and/or regions of tumor invasion with fewer number of cells, which tend to not exhibit EPR effect. However, more in-depth studies are required to prove this. It is well known that fluorescence intensity alone can vary widely patient-to-patient, owing to variability in fluorescent agent administration, delivery, and excretion, which increases variability causing the sensitivity and specificity to be diminished with selection of a population-based threshold for tumor vs. background associated level of fluorescence. The stability of PAI AUC of the ROC over patient populations with varied EGFR expression was likely due to the PAI ratiometric imaging methodology removing the hemodynamic variation of delivery and clearance rates of the dye among individuals. BP calculated using PAI could be a promising method for standardizing detection thresholds for tumor region detection, a hypothesis that will be explored in future planned clinical studies, and can be a significant component toward regulatory approval for using fluorescence to truly guide the surgeon’s actions.

Tumor spatial heterogeneity is an important prognostic factor, and accurately imaging receptor expression heterogeneity is key for identifying tumor regions. This is especially important when attempting to identify tumor in the surgical margins where cell density, and therefore EGFR concentrations, may be low. As anticipated from previous studies, PAI and ABY-029 were positively correlated, while untargeted IRDye 700DX was negatively correlated with EGFR IHC (Fig. 4) [22]. As image resolution decreases, Pearson’s coefficients of ABY-029 and PAI BP correlated with gold standard IHC image increased at a relatively constant rate (Fig. 4b). It can be observed that there is a large population of pixels in the IHC images that were clustered at the extreme measurable pixel values (Fig. 4a). This is likely due to the limited dynamic range of IHC images (0 ~ 2 OD). All three tumor lines had intense IHC staining that received a pathologist score of 3 + with strong, continuous membranous staining but were found to have nearly 2-orders of magnitude difference in EGFR expression determined by flow cytometry (1.2 (± 0.3) × 10^6^, 1.6 (± 0.6) × 10^5^, and 7.4 (± 0.4) × 10^4^ EGFR receptors/cell in A431, Detroit 562, and FaDu, respectively; see Supplemental Table S1 and Fig S7). The steady improvement of Pearson’s correlation can also reflect goodness of the registration between fresh tissue fluorescence images and fixed pathological tissue images, since imperfect registration could substantially reduce correlation and results in steep increase when lowering image resolutions to alleviate spatial misalignments. Interestingly, we had hypothesized that PAI would outperform SAI in measuring EGFR expression heterogeneity. While the variance in the correlation is much higher for SAI, both PAI and SAI were good predictors of EGFR expression heterogeneity.

Lower average variance of AUCs of PAI indicates PAI is a comparable imaging method among patients than SAI. Smaller standard deviation of variance of AUCs over time makes PAI a more stable and reliable signal during time course of surgery (Fig. 5c). Image contrast-to-variance (CVR) between the tumor and the normal tissue depends on many factors, including the administration-to-image timing (to allow normal tissue clearance), the instrumentation used, the dose of fluorophore given, the health of the patient (e.g., diseased liver/kidney may extend plasma half-life), the physiology of the tumor and healthy tissue (e.g., blood flow and vascular permeability), the on- and off-rate constants of specific agent binding, the level of nonspecific agent binding, and volume of tissue interrogated. In situ imaging, where decision-making may be most critical, tends to exhibit lower image contrast-to-variance than excised tissues [24], likely attributable to the nonspecific signal arising from the bulk normal tissue. There have been many strategies tested to increase contrast by decreasing nonspecific signal, including long administration-to-imaging times, administration of an unlabeled pre-dose, and the use of alternate imaging methodologies [25–27]. Improving tumor penetration of imaging agents can also increase contrast as demonstrated using a pre-dose to overcome the binding site barrier [27]. However, we have not observed any limitations in tumor penetration using ABY-029 or IRDye 700DX, as is observed for antibodies, so this is not likely the case for affibody molecules. Moreover, PAI does not address drug delivery as it only measures the concentration of receptors available to the imaging agent/drug but it has been demonstrated that PAI is capable of measuring changes in the available receptor concentration due to molecular therapeutics occupying receptors [28].

A large body of work, including clinical trials, has been produced using high-dose ICG and “second window” administration-to-imaging times (AIT), i.e., 24-h AIT. The high dose of ICG allows sufficient tumor accumulation such that the tumor is visible at 24 h, even with the fast plasma and normal tissue clearance typically observed [29]. In addition, several groups have demonstrated the use of IRDye 800CW labeled EGFR-targeted antibodies with surgery and imaging at 1–4 days post-administration [4, 5, 26, 27], with optimum fluorescence intensities observed within the first 2 days [26]. On the other hand, ABY-029 (~ 8 kD)—with its considerably faster plasma clearance half-life (~ 20 min) [6] as compared to monoclonal antibodies (~ 114 h)—yields optimal AIT of only a few hours [22, 30] as further demonstrated here. However, the ability of PAI to provide stable contrast and high tumor differentiation starting at 15 min (and extending for hours) highlights the potential for in-surgical suite administration of PAI, reducing complexities in patient appointments and surgical timing that can occur with SAI. Additionally, PAI is clinically feasible with FDA-approved Curadel Lab-Flare and Quest Spectrum for intraoperative imaging and Pearl and Odyssey CLx (Licor) back-table imaging [20].

In addition to PAI, AVB-620 is a protease-activated imaging agent which also uses dual-channel imaging [31]. In tumor tissues, protease-mediated hydrolysis of AVB-620 disrupts Förster energy transfer between Cy5 and Cy7, causing a large fluorescence ratio (FR) change. While this technique is less prone to optical artifacts compared to SAI, proteases are also present in normal tissues, which could cause non-tumor–specific change in FR to arise. Additionally, like other single-color activated fluorescence probes, there will be nonspecific fluorescence of the acceptor in both the normal and tumor tissues and issue in which PAI addresses. Also, dual-channel imaging has been applied on the quantification of drug delivery. Ian et al. developed a technique to quantify therapeutic protein distribution and degradation rates by labeling the protein with two dyes that demonstrate different residualization rates [32]. The ratio of the two is measured at the single cell level via ex vivo flow cytometry. This study depicts how protein therapeutics acting at the microscopic scale can further inform its tissue distribution and ultimate response. Finally, our collaborators developed TRIPODD (Therapeutic Response Imaging through Proteomic and Optical Drug Distribution) to evaluate in situ drug target availability with combination of paired-agent imaging and cyclic immunofluorescence [33]. The difference between TRIPODD with PAI presented in this study is that the imaging agents used are cell membrane permeable to achieve intracellular receptor imaging. This methodology is not intended for fluorescence-guided surgery but for therapeutic monitoring. All of these dual-channel methodologies are exciting examples of how quantitative fluorescence imaging can have a positive impact on clinical outcomes.

## Conclusions

PAI has the potential to broadly impact the clinical implementation of fluorescence-guided surgery by differentiating low and varied EGFR-expressing tongue SCCs with high accuracy and low inter-patient variance. PAI more accurately represented the true molecular heterogeneity of receptor expression in tumors over a wide range of clinically applicable resolutions. In addition, PAI demonstrated the potential to facilitate flexibility within the surgical setting by decreasing the time from imaging agent administration to the start of resection while maintaining high diagnostic accuracy. We propose the use of PAI as an innovative molecular imaging method that will improve the diagnostic accuracy and efficiency of FGS.

## Supplementary Material

Supplemental Material

## Figures and Tables

**Table 1.** Cohort statistics generated at the optimum ROC cut-off point

	FaDu			Detroit 562			A431			All Cell Lines		
	BP	ABY-029	IRDye 700	BP	ABY-029	IRDye 700	BP	ABY-029	IRDye 700	BP	ABY-029	IRDye 700
**Sensitivity**	0.903	0.872	0.306	0.929	0.932	0.766	0.881	0.889	0.774	0.898	0.809	0.661
**Specificity**	0.924	0.793	0.710	0.946	0.894	0.536	0.908	0.902	0.671	0.927	0.845	0.640
**Accuracy**	0.908	0.854	0.615	0.939	0.909	0.631	0.888	0.892	0.749	0.908	0.822	0.653
**PPV**	0.975	0.932	0.244	0.924	0.860	0.537	0.967	0.965	0.877	0.955	0.901	0.763
**NPV**	0.744	0.656	0.770	0.950	0.949	0.766	0.716	0.728	0.495	0.838	0.716	0.519

* The highest statistic parameter is highlighted in green

* *PPV* positive predictive value, *NPV* negative predictive value
