# Supplementary material for: Improved Discrimination of Tumors with Low and Heterogeneous EGFR Expression in Fluorescence-Guided Surgery Through Paired-Agent Protocols
Source: Mol Imaging Biol. 2021 Oct 14;25(1):110–21. doi: 10.1007/s11307-021-01656-3 (PMC9527767; doi:10.1007/s11307-021-01656-3)
Supplement: Supplementary file 1 — Supplementary file1 (PDF 38025 KB) [file 11307_2021_1656_MOESM1_ESM.pdf]

Supplemental Data

Improved discrimination of tumors with low and heterogeneous EGFR expression in fluorescence guided surgery through paired-agent protocols

S1. Effect of BSA binding and IRDye 700DX - ABY-029 binding

IRDye 700DX and ABY-029 were individually dissolved in PBS with or without 1% (w/v) bovine serum albumin (BSA) purchased from Sigma-Aldrich. The imaging agents, Chameleon® Duo fluorescent protein ladder (LI-COR), were loaded (7 µl) in 4-20% criterion TGX Stain-Free protein gel obtained from Bio-Rad. The gel was run for 40 min at 100 V, and then imaged with the Odyssey CLx (LI-COR) in 700- and 800-nm channels. Fluorescence quantification of each lane was performed in FIJI. The results showed 8.5 % ± 0.5 % of IRDye 700DX and 63.3 % ± 0.9 % of ABY-029 bound with BSA, while 0.6 % ± 0.3 % of IRDye 700DX bound with ABY-029.

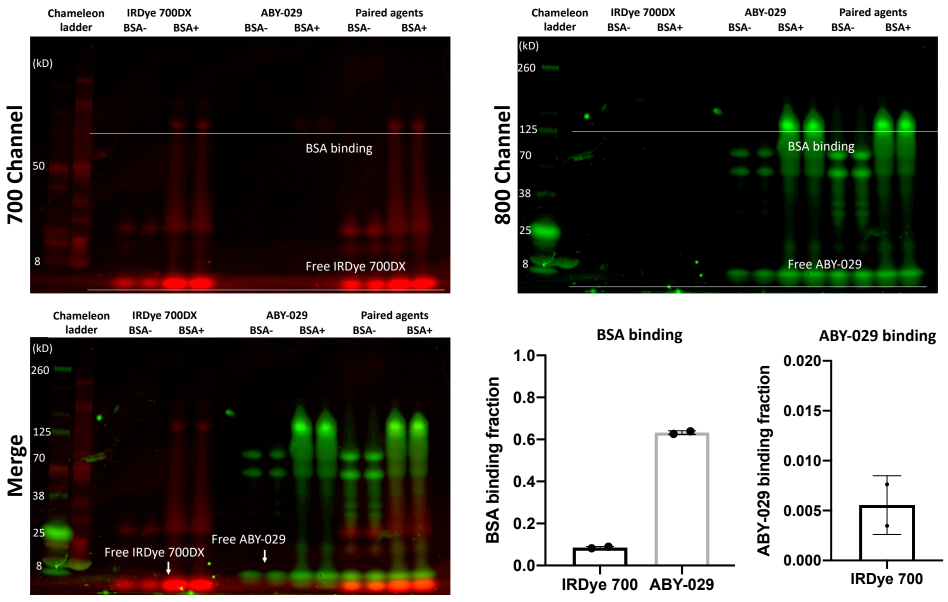

Figure S1. Gel electrophoresis for BSA-dye binding and IRDye 700DX-ABY-029 binding test.

S2. Determination of paired-agent dose based on autofluorescence signal

In order to determine the effect of autofluorescence on the observed fluorescence emission intensity, mice (n = 3, per cell line) bearing tongue tumors were sacrificed without injection of fluorescent agents. The tongues were bisected along the raphe and imaged on the Odyssey CLx scanner, as described for paired-agent injected mice in the *Methods*. The average fluorescence intensity of the tumor and surrounding normal tongue were calculated and compared to mice with a human-equivalent micro-dose (30 nanomoles) of each agent (n = 3). Using the method of Reagan-Shaw (2007) [1], the mouse equivalent dose was determined to be 48.8 μg/kg and an average mass of 22 g was used for a final dose of 1.07 μg per mouse. So, 10 x dose is 10.7 μg per mouse. At a micro-dose, the ABY-029 signal was > 15 times the autofluorescence signal in the 800-channel; however, the IRDye 700DX signal was < 5 times the 700-channel autofluorescence. Therefore, 10 times human-equivalent microdose (300 nanomoles) of IRDye 700DX was tested and at 3-hours post-administration it was found that the fluorescence signal was ~6-10 times that of the autofluorescence (Fig 2b & Fig S1b). Therefore, it was determined that ABY-029 and IRDye 700DX will be administered in a 1:10 molar ratio (30:300 nanomoles). This discrepancy in the dose of the PAI agents is accounted for in the normalization factor (Equation 2).

In addition to determining the dose of paired-agent administration, the autofluorescence values of each tissue affects the observed binding potential values. Typically, autofluorescence is subtracted off of every post-injection image - on a pixel-by-pixel basis if images are taken without moving the animal, or on average if the animal is moved between pre- and post-administration images. Here, it was found that pre-injection the BPs were -0.77 ± 0.03, -0.88 ± 0.004, -0.82 ± 0.008, and -0.83 ± 0.008 for normal tongue, FaDu, Detroit 562, and A431, respectively. The BP values for tumor are lower than that for normal tongue because the autofluorescence in the 700 nm channel

is stronger than that in the 800 nm channel. This indicates that the BP values we report here are actually underestimating the real BP when autofluorescence is considered, and the difference between normal and tumor tissue would actually be greater if the autofluorescence was considered.

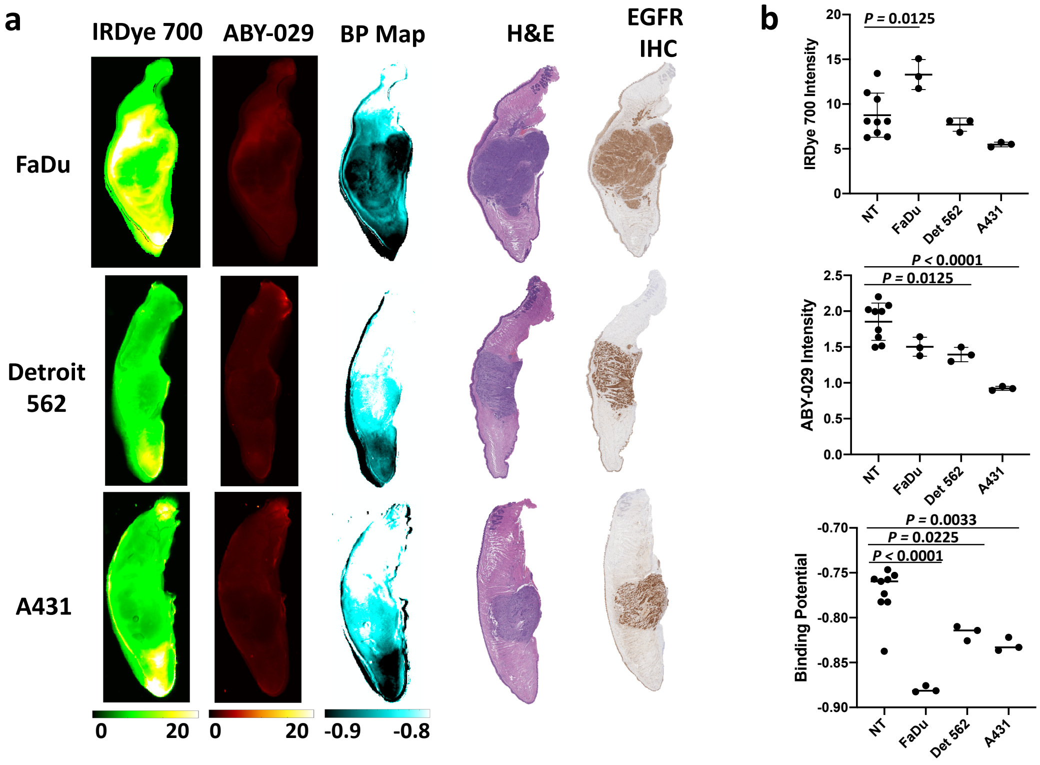

Figure S2. Determining doses of paired-agent imaging using auto-fluorescence. **(a)** For each tumor type, a representative example of the autofluorescence are shown for the 700 and 800 nm channels of the Odyssey CLx scanner and compared to standard H&E and epidermal growth factor receptor (EGFR) immunohistochemistry (IHC). **(b)** IRDye 700, ABY-029, and BP signals of each naïve tongue are shown in the corresponding box plot with mean and standard deviation.

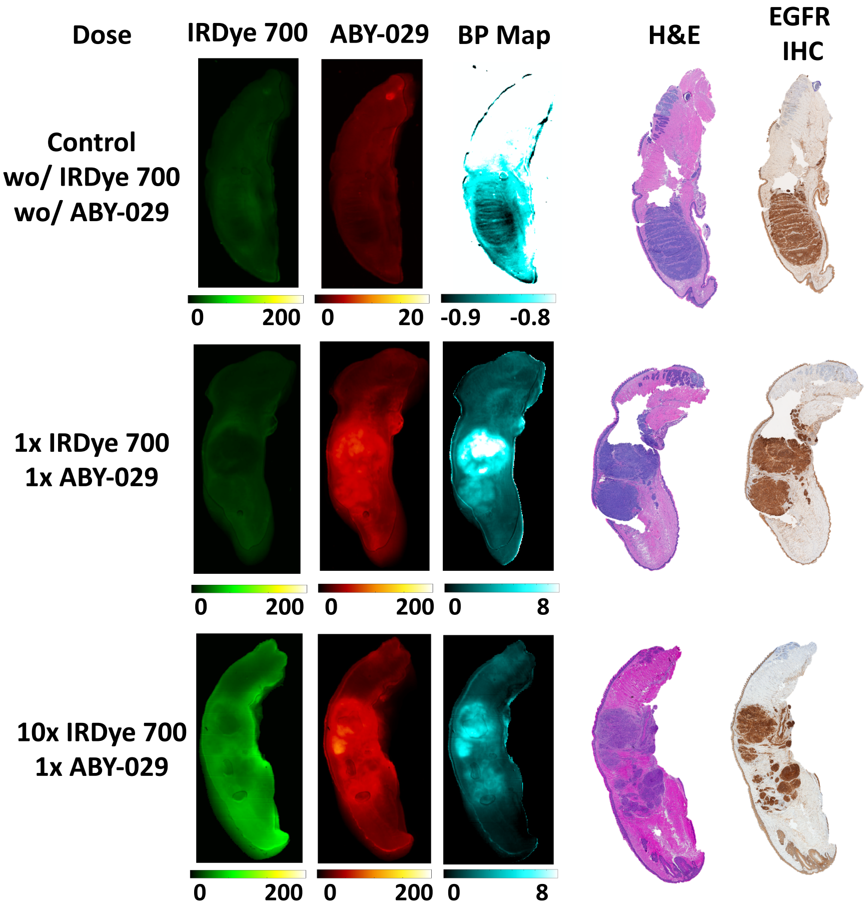

Figure S3. Fluorescent and pathological staining images are shown in 700 and 800 nm channels for auto-fluorescence, 1X dose of ABY-029 and IRDye 700DX (mouse equivalent to the human microdose, 30 nanomoles), and a 1X dose of ABY-029 and 10X dose of IRDye 700DX.

S3. Comparison of tumor-to-background ratio and contrast-to-variance ratio to measure image contrast

The normalization factor (NF) is an important part of calculating the binding potential (BP) as is corrects the fluorescence emission intensities of the targeted and non-targeted agent to remove effects of fluorescence emission efficiency and instrument excitation and collection efficiencies (Equation 2). Typically, the NF is calculated from tissue that lacks the receptor of interest (i.e.

muscle) on the first image post-administration of the paired-agents after autofluorescence subtraction. However, in this case and during surgery, it is nearly impossible to collect meaningful pre-injection images. To test the effect of the NF on the BP, the NF was varied from 0.2-0.4 and the resulting tumor-to-background ratio (TBR = average fluorescence in tumor divided by the average fluorescence in the normal tissue) and the contrast-to-variance ratio (CVR, Equation 3) was determined for each NF. As can be seen in Figure S2, varying the NF had an effect similar to window-leveling an image. The CVR remained stable at 1.7, while the TBR varied from 3.3 -19.0 over the range of NFs. As the NF increases in value, the fluorescence signal in the normal tissue decreases toward zero, causing an artificially high TBR. Although TBR is commonly used when analyzing contrast in FGS, it is an unstable measure as demonstrated here where the PAI determined BP could be artificially made to have high TBR values by simply altering the NF. CVR is a more robust measure that allows accurate comparisons between different imaging methods [2]. Note that the NF here is less than one because the IRDye 700DX is 10 times more concentrated than the ABY-029 for administration and therefore is brighter. The effect on TBR while changing the NF would be opposite if the targeted agent was brighter than the non-targeted agent.

For this study, the NF was determined in the tip of the tongue such that the resultant BP was equal to 0.5 (Equation 1). A BP of 0.5 was used instead of 0 in order to minimize pixels with a negative BP value in the normal tongue. For use during surgery, it is hypothesized that the surgeon could select a region of known normal tissue in order to determine the NF ratio for that patient. This would optimize visualization by creating a “zero background” signal in the normal surrounding tissues.

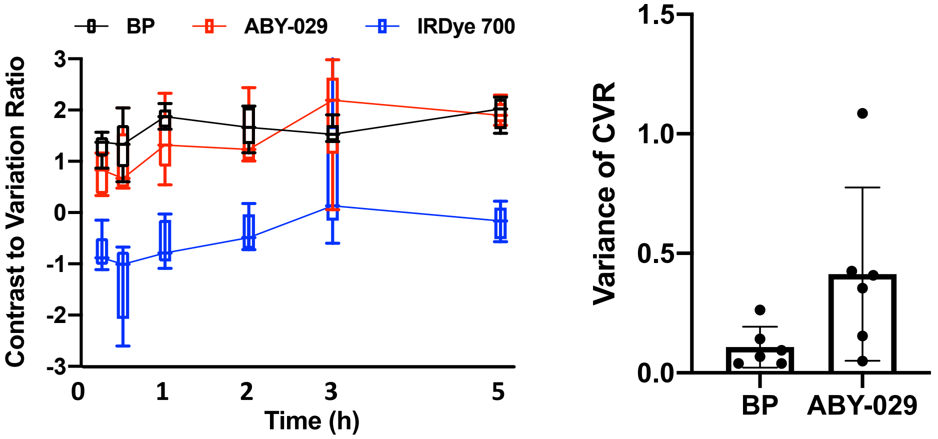

Figure S4. (**a**) PAI CVRs outperform ABY-029 SAI CVRs in most time points with less variance, suggesting that BP provides a stable diagnostic accuracy and tumor contrast starting at early administration-to-imaging time.

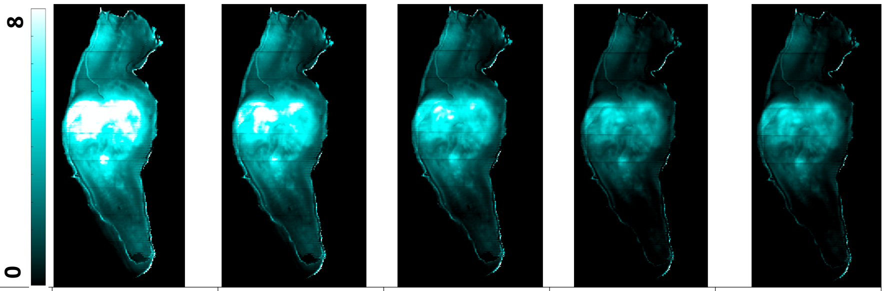

NR	0.2	0.25	0.3	0.35	0.4
CVR	1.7	1.7	1.7	1.7	1.7
TBR	3.3	3.9	5.0	7.5	19.0

Figure S5. The effect of the normalization factor (NF) on image contrast. As the normalization ratio increases, the overall signal decreases. This results in a constant contrast-to-variance ratio (CVR) that is stable when NF is varied. However, tumor-to-background ratio (TBR) increases as

the signal in the normal tissue (the denominator) decreases. These results indicate that TBR is an unstable measure for binding potential and cannot be used to compare to single agent fluorescence.

S4. Spatial alignment of fresh tissue sections with pathological sections

Assessment of the spatial alignment performed between the IHC and the fluorescence images was performed by creating image overlays with the *mshowpair* function in Matlab (Figure S3) [3]. "Checkerboard" creates an image with alternating rectangular regions from fluorescence and IHC images.

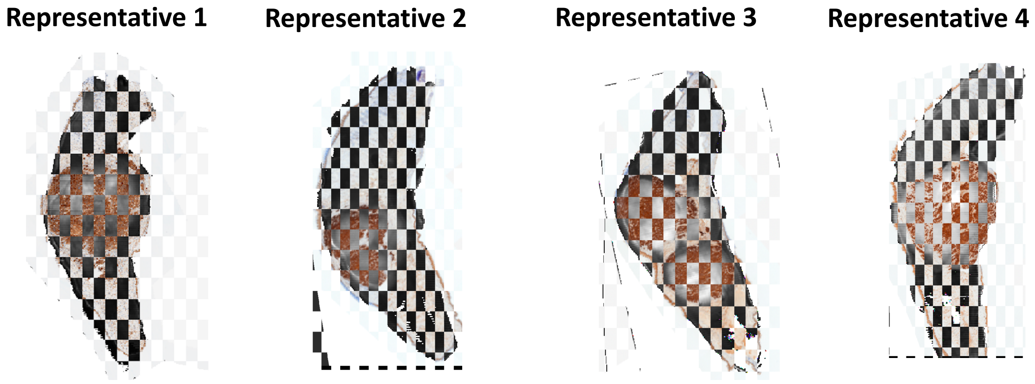

Figure S6. Image overlays created using *imshowpair* in Matlab allow visual assessment of spatial alignment between IHC and BP images. In the Checkerboard overlay, IHC is in RGB (brown pixels) while the BP map is a grey scale image.

S5. Determination of EGFR per cell using flow cytometry

The methodology for the quantification of the number of EGFR per cell using flow cytometry has been described previously in detail [4]. Briefly, cells were purchased from and cultured as per instructions from ATCC. Trypsinized and washed cells (5×10^5) were labeled with either 4 mg/ml of EGF Biotin (Molecular Probes, Invitrogen, Camarillo, CA, USA) or PBS in triplicate for each trial. All cells were secondarily labelled with 1:25 dilution of Cy-5 Streptavidin (Invitrogen,

Camarillo, CA, USA. Flow cytometric data was acquired with a FACSCalibur analysis system equipped with a FACStation, and Cell Quest Acquisition software (Becton Dickinson, San Jose, CA, USA). The results compared to Cy5 Quantum Cy5 MESF beads by creating a standard fluorescence concentration curve using QuickCal v2.3 software as provided by the manufacturer (Bangs Laboratory, Fishers, IN, USA). The geometric mean of each sample was used to determine the concentration of fluorophore from the concentration curve and the average value of EGF stained cells and control cells are reported in Table S1. The average control signal was subtracted from the average EGF stained cells to remove the contribution of autofluorescence and non-specific staining. Finally, the receptors per cell was calculated assuming one molecule of biotin per EGF and three Cy5 fluorophores per streptavidin molecule, as specified by Invitrogen. The entire procedure was repeated three times for each cell line, with the exception of A431, which was repeated four times due to large variation. The values for the EGF stained and control cells for each cell line are summarized in Table S1 and the average value plotted in Figure S5.

The cell lines selected for the study were A431, Detroit 562, and FaDu based on a combination of the EGFR expression (high, moderate, low-moderate HNSCC expressing, respectively) and the ability to successfully grow in orthotopic tongue tumors in mice. Comparatively, it is reported that normal epithelial cells have ~$5x10^3$ EGFR per cell, while other normal cells (such as hepatocytes) can have as many as $2x10^5$ EGFR per cell [5]. Here, our cell lines were determined to have $3.0x10^4$ – $1.2x10^6$ EGFR per cell, indicating a wide range of expression levels. In 1998, Grandis et al. compared EGFR expression in 91 patient HNSCC samples to A431 positive control cell line, and found that the median expression was 54% of the A431 standard with a range of 5-233%. Here, we determined that FaDu has 6.2% the EGFR per cell as compared to A431, indicating that our FaDu results represent a low EGFR expressing HNSCC [6].

Table S1. Summary of individual trial and average values of EGFR per cell determined by quantitative flow cytometry

		Squamous Cell Carcinoma Cell Lines					
		**A431**	**SCC-15**	**SCC-25**	**Detroit 562**	**FaDu**	**SCC-9**
**Trial 1**	EGF Stained	4440540	1787549	1113122	593638	307525	210564
	Control	46448	99511	135942	8875	76920	115359
	EGF Stained - Control	4394092	1688039	977181	584763	230604	95205
	**EGFR/cell**	**1464697**	**562680**	**325727**	**194921**	**76868**	**31735**
**Trial 2**	EGF Stained	4224933	1625443	1251095	571489	231411	95078
	Control	53591	9653	48738	10691	5562	8774
	EGF Stained - Control	41713412	1615790	1202357	560798	225849	86304
	**EGFR/cell**	**1390447**	**538597**	**400786**	**186933**	**75283**	**28768**
**Trial 3**	EGF Stained	2898956	1652385	1071946	268314	212193	92306
	Control	57527	7181	9252	4332	2791	7400
	EGF Stained - Control	2841429	1645204	1062694	263982	209402	84906
	**EGFR/cell**	**947143**	**548401**	**354231**	**87994**	**69801**	**28302**
**Trial 4**	EGF Stained	3040764	-	-	-	-	-
	Control	44117	-	-	-	-	-
	EGF Stained - Control	2996647	-	-	-	-	-
	**EGFR/cell**	**998882**	**-**	**-**	**-**	**-**	**-**
**EGFR /Cell**	**Average**	**120×10^4**	**55×10^4**	**36×10^4**	**16×10^4**	**7.4×10^4**	**3.0×10^4**
	**Std. Dev.**	**30×10^4**	**1×10^4**	**4×10^4**	**6×10^4**	**0.4×10^4**	**0.2×10^4**

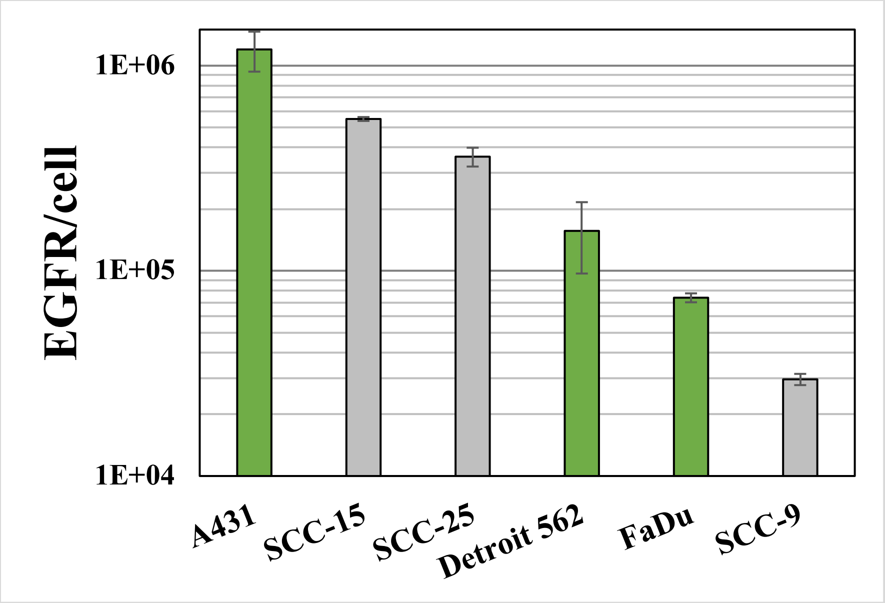

Figure S7. EGFR molecules per cell line for squamous cell carcinomas determined by quantitative flow cytometry. Cell lines used for this study are indicated in green.

Supplementary References

1. Reagan-Shaw S, Nihal M, Ahmad N (2008) Dose translation from animal to human studies revisited. FASEB J 22:659–661. https://doi.org/10.1096/fj.07-9574LSF

2. Tichauer KM, Wang C, Xu X, Samkoe KS (2020) Task-based evaluation of fluorescent-guided cancer surgery as a means of identifying optimal imaging agent properties in the context of variability in tumor- and healthy-tissue physiology. In: Molecular-Guided Surgery: Molecules, Devices, and Applications VI. International Society for Optics and Photonics, p 112220O

3. Muthukumaran D, Sivakumar M (2017) Medical Image Registration: A Matlab Based Approach

4. Tichauer KM, Samkoe KS, Sexton KJ, et al (2012) In vivo quantification of tumor receptor binding potential with dual-reporter molecular imaging. Molecular Imaging and Biology 14:584–592

5. Quantitative Analysis of Endocytosis and Turnover of Epidermal Growth Factor (EGF) and EGF Receptor - Sorkin - 2010 - Current Protocols in Cell Biology - Wiley Online Library. https://currentprotocols.onlinelibrary.wiley.com/doi/abs/10.1002/0471143030.cb1514s46. Accessed 12 May 2021

6. Grandis JR, Melhem MF, Gooding WE, et al (1998) Levels of TGF-α and EGFR Protein in Head and Neck Squamous Cell Carcinoma and Patient Survival. Journal of the National Cancer Institute 90:824–832. https://doi.org/10.1093/jnci/90.11.824
